# Caveolin 1 protein expression in renal cell carcinoma predicts survival

**DOI:** 10.1186/1471-2490-11-25

**Published:** 2011-12-07

**Authors:** Sandra Steffens, Andres J Schrader, Hanna Blasig, Gesa Vetter, Hendrik Eggers, Wolfgang Tränkenschuh, Markus A Kuczyk, Jürgen Serth

**Affiliations:** 1 Department of Urology, Hannover Medical School, (Carl-Neuberg-Strasse 1), Hannover, (30625), Germany; 2 Department of Urology, Ulm-University Medical School, (Prittwitzstrasse 43), Ulm, (89075), Germany; 3 Department of Pathology, Hannover Medical School, (Carl-Neuberg-Strasse 1), Hannover, (30625), Germany

## Abstract

**Background:**

Caveolae play a significant role in disease phenotypes such as cancer, diabetes, bladder dysfunction, and muscular dystrophy. The aim of this study was to elucidate the caveolin-1 *(*CAV1*) *protein expression in renal cell cancer (RCC) and to determine its potential prognostic relevance.

**Methods:**

289 clear cell RCC tissue specimens were collected from patients undergoing surgery for renal tumors. Both cytoplasmic and membranous CAV1 expression were determined by immunohistochemistry and correlated with clinical variables. Survival analysis was carried out for 169 evaluable patients with a median follow up of 80.5 months (interquartile range (IQR), 24.5 - 131.7 months).

**Results:**

A high CAV1 expression in the tumor cell cytoplasm was significantly associated with male sex (p = 0.04), a positive nodal status (p = 0.04), and poor tumor differentiation (p = 0.04). In contrast, a higher than average (i.e. > median) CAV1 expression in tumor cell membranes was only linked to male sex (p = 0.03). Kaplan-Meier analysis disclosed significant differences in 5-year overall (51.4 vs. 75.2%, p = 0.001) and tumor specific survival (55.3 vs. 80.1%, p = 0.001) for patients with higher and lower than average cytoplasmic CAV1 expression levels, respectively. Applying multivariable Cox regression analysis a high CAV1 protein expression level in the tumor cell cytoplasm could be identified as an independent poor prognostic marker of both overall (p = 0.02) and tumor specific survival (p = 0.03) in clear cell RCC patients.

**Conclusion:**

Over expression of caveolin-1 in the tumour cell cytoplasm predicts a poor prognosis of patients with clear cell RCC. CAV1 is likely to be a useful prognostic marker and may play an important role in tumour progression. Therefore, our data encourage further investigations to enlighten the role of CAV1 and its function as diagnostic and prognostic marker in serum and/or urine of RCC patients.

## Background

Renal cell carcinoma (RCC) is a common urologic tumor and accounts for about 3% of all human malignancies. A significant increase in its incidence has been observed during the last decades, and the annual mortality-to-incidence ratio for RCC is considerably higher than for other tumors of the genitourinary tract [1].

Tumor characteristics such as tumor stage and grade seem to have limited value in predicting the clinical outcome of individual patients as around 50% of patients who undergo surgery with curative intent for less advanced disease can be expected to develop a distant recurrence. Moreover, RCC encompasses many histological subtypes with distinct genetic and biologic features that determine clinical course and outcome [2]. Therefore, an increased understanding of genetic and biologic changes could help to develop a valuable marker to improve the individual therapeutic management and clinical outcome of RCC.

An essential step in the formation of metastases is the invasion of tumor cells into the extra cellular matrix. Cell adhesion molecules and extra-cellular matrix proteins can either support an increase or a decrease in the ability of tumor cells to adhere to surrounding tissue. Caveolin-1 *(*CAV1*) *has been identified two decades ago; it has been proposed to act as a tumor suppressor protein, inhibiting the functional signaling activity of several proto-oncogenes and consequently disrupting the process of cellular transformation [3-12]. Numerous follow-up studies designed to test this hypothesis have contributed a myriad of evidence suggesting that CAV1 may indeed possess tumor suppressor capabilities. For instance, CAV1 mRNA and protein expressions are down regulated in NIH-3T3 cells transformed with several activated oncogenes, such as v-Abl, Bcr-Abl, and H-Ras (G12V) [3,9]. Genetic evidence supporting the role of CAV1 as a tumor suppressor has emerged from gene mapping studies, which revealed that the human CAV-1 gene maps to the long arm of human chromosome 7 (7q31.1). However, a number of clinicopathologial studies have shown a positive correlation between CAV1 over expression and advanced renal cell cancer, metastasis and poor prognosis [13]. In addition, these studies yielded variable and even contradicting results in terms of over expression in different histological subtypes [2].

The aim of this study was to elucidate the expression of CAV1 in RCC and to determine its potential prognostic relevance for patients with clear cell cancer.

## Methods

### Tissue specimens

The present study included 289 patients, who underwent radical nephrectomy between 1979 and 1998 in the Hannover Medical School. The ethical committee of the institution approved the study. Tissue was obtained from archival routine surgical specimens. The tissue samples were selected by a pathologist and prepared from the primary tumor and arranged on tissue micro arrays (TMA) as described previously [14]. Two pathologists evaluated all specimens with respect to tumor stage, grade, and histological subtypes. Tumour samples were classified primarily according to UICC 1997 TNM tumour staging system [15] and nuclear grading was based on the Fuhrman grading system [16]. Histological subtypes were assessed according to the consensus classification of renal cell neoplasia [17]. Data were collected by physicians and data managers and subsequently maintained by a relational database.

### Patients

The median age of the cohort was 60.4 years (SD ± 11.3 months). 159 patients were men (55%), and 130 patients were women (45%). 8, 150, 115, and 16 presented with pT1, pT2, pT3 and pT4 cancer, respectively. Tumour differentiation showed that 50, 181, 21 suffered from G1, G2, and G3/4 tumours. Furthermore, 29 patients presented with lymph node and 55 patients with visceral metastasis.

In the majority of cases, data regarding the cancer-specific long-term survival (CSS) were retrieved from electronic patient charts. The duration of the follow-up was calculated from date of surgery to the date of death or last follow-up. Death was assessed as either cancer-related or unrelated. Survival analysis was carried out for 169 evaluable patients with complete follow-up data and pathologically proven clear cell carcinoma of the kidney. The follow up group (n = 169) exhibited a median follow-up period of 80.5 months (IQR, 24.5 - 131.7 months). At the time of the last follow-up examination, 80 of patients were alive, 63 patients had died from progressive RCC and 26 patients due to other causes.

### Procedures

Expression of CAV1 was determined by immunohistochemistry (IHC). The paraffin-embedded TMA samples were deparaffinized, rehydrated and immersed in 3% hydrogen peroxide solution to block endogenous peroxidase activity. Antigen retrieval was accomplished by microwave heating specimens in a 0.01 M citrate buffer for 15 min. Biomarker expression was immunohistochemically detected by commercially available antibodies (CAV1 rabbit polyclonal anti-caveolin-1 dilution 1:100, Becton Dickinson Biosciences, Franklin Lakes, NJ, USA). After 12 h of incubation the sections were washed in TBS and incubated with a secondary biotinylated antibody (Vectastatin Elite ABC Kit, Vector Laboratories, Inc., Burlingame, CA, USA) for 60 min. and visualization using the DAB systems according to the manufacturer's instructions. Sections were briefly rinsed in tap water, counterstained with Mayer's Haematoxylin solution and then mounted. For negative control, the primary antibody was replaced by non-immune serum. All tissue staining were assessed in a blind study by two independent investigators (H.B and S.W.).

The rabbit polyclonal anti-caveolin-1 from Becton Dickinson Biosciences (Franklin Lakes, NJ, USA) recognises both the a and b isoforms of caveolin-1 as assessed by Western blotting. Negative controls run in parallel-comprised sections where the primary antibody had been omitted. Caveolin-1 staining of peripheral endothelial cells and non-neoplastic tissue adjacent to the tumour were used as the positive controls (also see additional file 1, figure S1). In negative controls the primary antibody was omitted.

The expression of CAV1 was evaluated in the membrane, cytoplasm and nucleus of the tumour cells. The staining reaction was classified according to a semi-quantitative IHC reference scale as previously described [18-22]. Membranous CAV1 expression was scored using the internal vascular endothelial cells as the positive control. Staining stronger than, equivalent to, or weaker than the vascular endothelial cells was scored as 3, 2 and 1, respectively, and the absence of staining was scored as 0 [13] (also see additional file 2, figure S2). In addition, cytoplasmic CAV1 staining stronger than, equivalent to, or weaker than the vascular endothelial cells was scored as 5, 3 and 1, respectively; and the absence of staining was scored as 0. Furthermore, the scores 4 and 2 describing staining partly classified as 5 and 3 and 3 and 1, respectively, were added. Adding the stained area (1 = 0-5%, 2 = 6-25%, 3 = 26-50%, 4 = 51-75% and 5 = 76-100%) a novel staining intensity score was defined by multiplying the score with the stained area. Given the absence of normative data on cell membrane or cell cytoplasma staining intensity in the literature, values in our patient collective were dichotomized using the median of observed distribution as the cut off.

### Statistical analysis

The primary endpoints of this study were tumor specific and overall-survival. Continuous variables were reported as means and standard deviations (SD) for parametric distributions or as medians and interquartile ranges (IQRs) for nonparametric distributions. Kaplan-Meier survival times were calculated, and subgroups were compared by the log-rank test statistic. Multivariate Cox regression models were used to assess the association between survival and cell membrane as well as cytoplasmic CAV1 expression adjusted for different clinical and patient covariates. The chi-square test and Fisher's exact test were conducted to assess associations between cell membrane/cytoplasm and patient/tumor specific characteristics. SPSS 17.0 was used for statistical assessment. P values below 0.05 were considered significant in all tests. All p values were two-sided.

## Results

### Correlation between patient characteristics and CAV1 expression

CAV1 expression was detected in the tumor cell cytoplasm of 242 (83.7%) and the tumor cell membrane of 232 (80.3%) patients with clear cell RCC. The median staining intensity was 3 (range: 1-25, IQR: 1-9) for tumor cell cytoplasm, and 3 (range: 1-15, IQR: 1-6) for tumor cell membranes, respectively. CAV1 protein expression in the tumor cell cytoplasm and cell membrane correlated moderately but significantly (r = 0.52, p < 0.001, Pearson). The cell nuclei were CAV1 negative in all patients' tumor specimens.

A high CAV1 expression in the tumor cell cytoplasm was significantly associated with male sex (p = 0.044), a positive nodal status (p = 0.042), and poor tumor differentiation (p = 0.035; table 1). In contrast, a higher than average (i.e. > median or > 3 intensity score) CAV1 expression in tumor cell membranes was only linked to male sex (p = 0.03; table 2). There was no significant correlation between CAV1 staining and patient age, tumor stage, and visceral metastasis.

**Table 1:** Association of different patient and tumor specific characteristics with CAV1 protein expression in the tumor cytoplasm.

Variable	CAV1 in tumor cytoplasm ≤ median	CAV1 in tumor cytoplasm > median	p-value	Test
Age (mean; ± SD)	60.4 ± 11.5 years	59.3 ± 11.1 years	0.40	t-test
Sex			0.04	Fisher's exact
female	79 (50.6%)	51 (38.3%)		
male	77 (49.4%)	82 (61.7%)		
Stage (TNM 2002)			0.54	Chi^2^
pT1	5 (3.2%)	3 (2.3%)		
pT2	86 (55.1%)	64 (48.1%)		
pT3	58 (37.2%)	57 (42.9%)		
pT4	7 (4.5%)	9 (6.7%)		
LN metastasis^1^			0.04	Fisher's exact
pN0	78 (89.7%)	70 (77.8%)		
pN+	9 (10.3%)	20 (22.2%)		
Pulmonal/visceral metastasis^1^			0.23	Fisher's exact
M0	124 (82.7%)	93 (76.2%)		
M+	26 (17.3%)	29 (23.8%)		
Grade			0.04	Chi^2^
G1	30 (21.6%)	20 (17.7%)		
G2	103 (74.1%)	78 (69.0%)		
G3/4	6 (4.3%)	15 (13.3%)		

^1 ^at time of renal surgery.

Abbreviations: CAV1 = caveolin 1, RCC = renal cell carcinoma, SD = standard deviation, LN = lymph node.

**Table 2:** Association of different patient and tumor specific characteristics with CAV1 protein expression in tumor membranes.

Variable	CAV1 in tumor membranes ≤ median	CAV1 in tumor membranes > median	p-value	Test
Age (mean; ± SD)	59.9 ± 11.7 years	59.8 ± 10.8 years	0.99	t-test
Sex			0.03	Fisher's exact
female	87 (50.3%)	43 (37.1%)		
male	86 (49.7%)	73 (62.9%)		
Stage (TNM 2002)			0.54	Chi^2^
pT1	4 (2.3%)	4 (3.4%)		
pT2	91 (52.6%)	59 (50.9%)		
pT3	66 (38.2%)	49 (42.2%)		
pT4	12 (6.9%)	4 (3.4%)		
LN metastasis^1^			1.00	Fisher's exact
pN0	86 (83.5%)	62 (83.8%)		
pN+	17 (16.5%)	12 (16.2%)		
Pulmonal/visceral metastasis^1^			0.54	Fisher's exact
M0	133 (81.1%)	84 (77.8%)		
M+	31 (18.9%)	24 (22.2%)		
Grade			0.28	Chi^2^
G1	30 (20.2%)	20 (19.4%)		
G2	110 (73.8%)	71 (68.9%)		
G3/4	9 (6.0%)	12 (11.7%)		

^1 ^at time of renal surgery.

Abbreviations: CAV1 = caveolin 1, RCC = renal cell carcinoma, SD = standard deviation, LN = lymph node.

### CAV1 expression predicts the clinical course

The calculated median five-year overall and tumor specific survival ratio of all 169 evaluable patients was 65.0% and 69.5% months, respectively.

**Figure 1:**
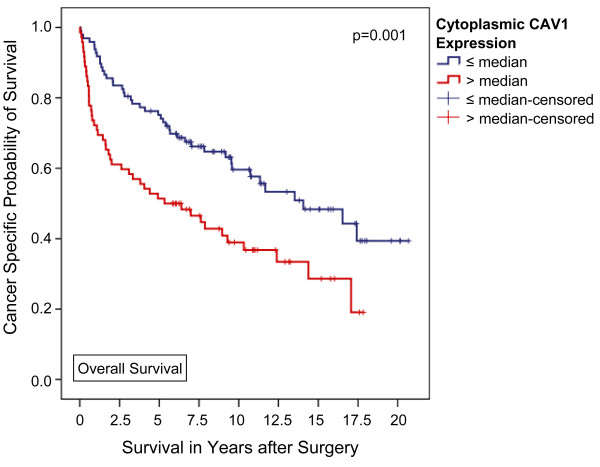
**Association between cytoplasmic CAV1 expression and clinical outcome in all patients (Kaplan-Meier; n = 169): The overall survival of patients with a higher-than-average CAV1 expression in the tumor cell cytoplasm was significantly shorter compared with that of patients lower CAV1 levels**. 5-year survival rates were calculated at 51.4% and 75.2% (p = 0.001, Mantel-Cox).

**Figure 2:**
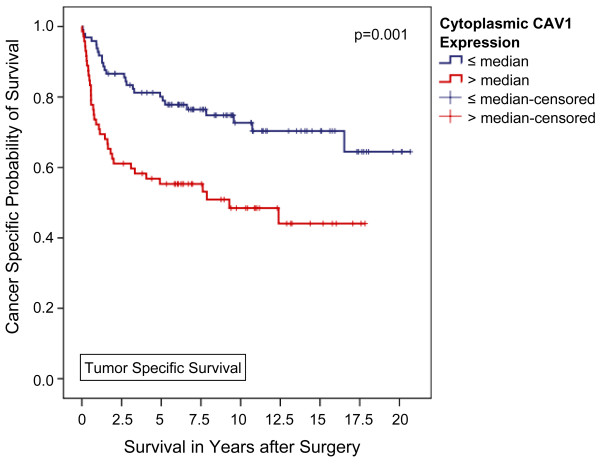
**Association between cytoplasmic CAV1 expression and clinical outcome in all patients (Kaplan-Meier; n = 169): The tumor-specific survival of patients with a higher-than-average CAV1 expression in the tumor cell cytoplasm was significantly shorter compared with that of patients lower CAV1 levels**. 5-year survival rates were calculated at 55.3% and 80.1% (p = 0.001, Mantel-Cox).

With a median follow-up of 80.5 months (IQR, 24.5 - 131.7 months), a higher disease-related death rate was observed among patients with higher-than-average cytoplasmic CAV1 levels (50.7% vs. 26.8%, p = 0.002, Fisher's exact test). Moreover, Kaplan-Meier analysis disclosed significant differences in overall and tumor specific survival for patients with higher and lower than average cytoplasmic CAV1 expression levels, respectively. The calculated 5-year survival rates for patients with high vs. low cytoplasmic CAV1 levels (i.e. staining intensity 0-3 vs. ≥4) were 51.4% vs. 75.2% for overall survival (p = 0.001, log rank) and 55.3% vs. 80.1% for disease specific survival (p = 0.001, log rank), respectively (Figures 1 and 2). Furthermore, tumor-specific survival of patients with a CAV1 staining score of 4 or 5 (n = 19) in the tumor cell cytoplasm was significantly shorter compared with that of patients with a score of 2-3 (n = 38) and even more 0-1 (n = 112) with 5-year tumor specific survival rate of 26.3%, 60.0%, and 80.1%, respectively (p < 0.001, Mantel-Cox; additional file 3, figure S3).

**Figure 3:**
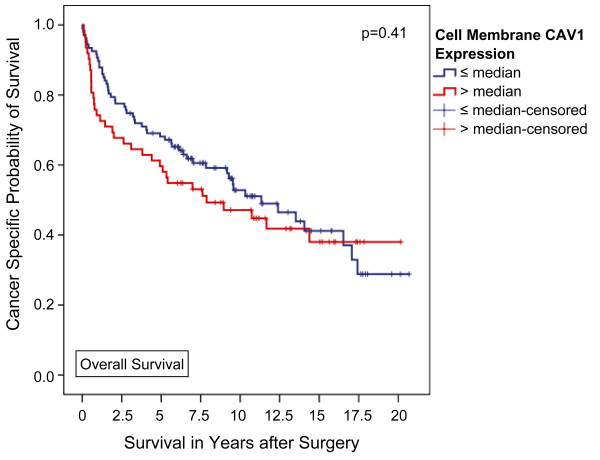
**Association between cell membrane CAV1 expression and clinical outcome in all patients (Kaplan-Meier; n = 169): The overall survival of patients with a higher-than-average CAV1 expression in the tumor cell membrane did not significantly differ from that of patients lower CAV1 levels**. 5-year survival rates were calculated at 59.7% and 68.1% (p = 0.41, Mantel-Cox).

**Figure 4:**
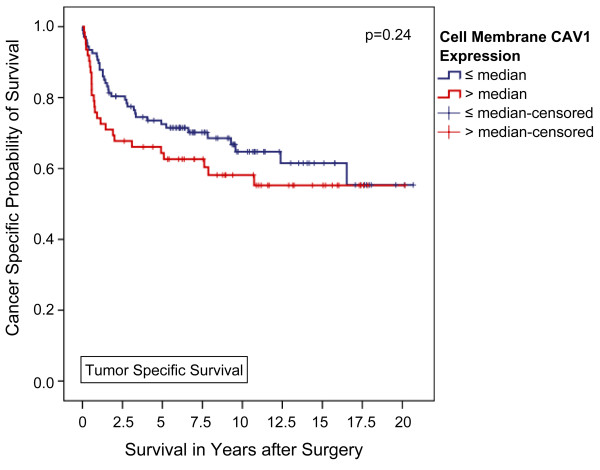
**Association between cell membrane CAV1 expression and clinical outcome in all patients (Kaplan-Meier; n = 169): The tumor-specific survival of patients with a higher-than-average CAV1 expression in the tumor cell membrane did not significantly differ from that of patients lower CAV1 levels**. 5-year survival rates were calculated at 64.4% and 72.5% (p = 0.24, Mantel-Cox).

In contrast, using univariate analysis, high membranous CAV1 failed to significantly predict tumor associated death rates during follow up (42.9% vs. 33.6%, p = 0.25, Fisher's exact test). Accordingly, neither overall nor tumor specific Kaplan-Meier survival were significantly associated with CAV1 expression located in the tumor cell membranes (p = 0.41 and 0.24, respectively; Figures 3 and 4). Furthermore, tumor-specific survival of patients with a CAV1 staining score of 2 or 3 (n = 50) in the tumor cell membrane was slightly but insignificantly shorter compared with that of patients with a score of 1 (n = 85) or 0 (n = 34) with a 5-year tumor specific survival rate of 61.9%, 72.8%, and 72.7%, respectively (p = 0.56, Mantel-Cox; additional file 4, figure S4).

Applying multivariable Cox regression analysis, including age, sex, stage, metastasis status, and tumor grade, in contrast to CAV1 cell membrane expression (p = 0.51; HR 1.17, 95% CI 0.73-1.87, and p = 0.34; HR 1.31, 95% CI 0.75-2.30), a high CAV1 protein expression level in the tumor cell cytoplasm could be identified as an independent poor prognostic marker of both overall (p = 0.022; HR 1,74, 95% CI 1.08-2.80) and tumor specific survival (p = 0.026; HR 1.95, 95% CI 1.08-3.51) in RCC patients (table 3).

**Table 3:** Multivariable Cox regression analysis identified an elevated CAV1 staining value in the tumor cytoplasm as an independent predictor of tumor specific survival.

	CAV1 expression in the tumor cell cytoplasm		CAV1 expression in the tumor cell membrane	
**Variable**	**HR (95% CI)**	**p-value**	**HR (95% CI)**	**p-value**

Age	1.01 (0.99-1.01)	0.35	1.01 (0.98-1.03)	0.71
Sex		0.45		0.44
female	1 (Reference)		1 (Reference)	
male	1.26 (0.70-2.28)		1.27 (0.69-2.23)	
T-Stage		0.43		0.19
pT1	1 (Reference)		1 (Reference)	
pT2	1.43 (0.19-10.86)	0.73	1.43 (0.19-10.92)	0.73
pT3	2.28 (0.30-17.58)	0.43	2.64 (0.35-20.28)	0.35
pT4	2.97 (0.27-33.25)	0.38	3.39 (0.30-37.89)	0.32
Metastatic disease		< 0.001		< 0.001
N/M0	1 (Reference)		1 (Reference)	
N/M+	5.04 (2.66-9.54)		4.88 (2.60-9.16)	
Differentiation		< 0.001		< 0.001
G1	1 (Reference)		1 (Reference)	
G2	2.75 (1.07-7.10)	0.04	2.55 (0.99-6.55)	0.05
G3/4	12.02 (3.54-40.85)	< 0.001	12.29 (3.60-41.98)	< 0.001
CAV1 expression level		0.03		0.34
≤ median	1 (Reference)		1 (Reference)	
> median	1.95 (1.08-3.51)		1.31 (0.75-2.30)	

## Discussion

Increased CAV1 expression has been reported to be associated with progression of papillary carcinoma of the thyroid, high-grade bladder cancer, poor prognosis of pancreas cancer, and lymph node metastasis in esophageal squamous cell carcinoma. Moreover, increased CAV1 expression has been reported to be associated with various pathological parameters, including higher Gleason score in prostate cancer, lymph node metastasis and positive surgical margins, and it has been shown to be an independent prognostic marker for progression in clinically localized prostate cancer [23]. These studies indicate that CAV1 functions as a tumor metastasis and progression-promoting molecule. On the other hand, CAV1 has also been implicated in the inhibition of cancer progression. For example, CAV1 expression is frequently lost in colon cancer, ovarian cancer, lung cancer and sarcoma [9,24-27]. Therefore, the physiological role of CAV1 in cancer cells is quite complicated depending e.g. on the type of cancer and the tumor origin. Several studies using tissue microarry (TMA) and immunostaining have shown similar associations between the increased expression of CAV1 and clinicopathological parameters in RCC as described in prostate cancer. A major difference between prostate and kidney tissue is that CAV1 is present at high levels in normal kidney tissue independent of CAV1 levels in tumor tissue but not in normal prostate tissue [23].

Several groups of investigators have studied the prognostic significance of caveolin-1 protein expression. Horiguchi et al. [28] reported that in clear cell RCC patients increased caveolin-1 expressions also correlated with tumor aggressiveness. Campbell et al. [13] also described that higher caveolin-1 expression was correlated with shorter survival in 69 patients with clear cell histology.

Previously, we were able to show that *CAV1 *mRNA expression is higher in RCC compared to normal renal tissue and increases with tumour stage [29]. In the present study, which until today is the largest study cohort with a sufficient follow-up period, Caveolin 1 protein expression was correlated with clinico-pathological parameters and survival in patients with clear cell RCC. Kaplan-Meier analysis disclosed significant differences in overall and tumor specific 5-year survival for patients with higher and lower than average cytoplasmic CAV1 expression levels, i.e. 51.4% vs. 75.2% for overall survival and 55.3% vs. 80.1% for disease specific survival, respectively. This supports the hypothesis that CAV1 plays a potential role in renal carcinogenesis or at least RCC progression. In contrast, a study by Tamaskar et al. with 22 RCC patients there was no correlation between membranous or cytoplasmic caveolin-1 expression and other clinical parameters, with membranous caveolin-1 expression being detected predominantly in clear cell RCC. Mete et al. [30] studied 112 renal tumors with different histological subtypes also using polyclonal rabbit antihuman CAV1 antibody and observed that staining was mainly cytoplasmic in all tumor groups. Also Campbell et al. [13] showed in that staining is predominantly found in the tumor cell cytoplasm of RCC patients. We were able to confirm these results as CAV1 expression was detected in the tumor cell cytoplasm of 242 (83.7%) and the cell membrane of 232 (80.3%) patients with clear cell RCC, the cell nuclei were negative in all patients' tumor specimens. Furthermore, high CAV1 protein expression level in the tumor cell cytoplasm could be identified as an independent poor prognostic marker.

The reasons for these findings are still unclear, as CAV1 expression would be expected to be mainly found on the cell surface. Tahir et al. [31] where able to show that in prostate cancer cell lines CAV1 is secreted in response to androgens and glucocorticoids leading to survival and clonal growth of these cells and thereby contributing to their metastatic potential and androgen insensitivity. Tahir et al was able to show that elevated preoperative levels of serum CAV1 predicts decreased time to cancer recurrence [32]. Adapted from these results, one explanation for the cytoplasmatic expression of CAV1 in clear cell RCC might be that within the transformed cells, CAV1 is rerouted into the secretory pathway of these cells, and that the cytoplasmatic CAV1 accumulation may contribute to the transformed phenotype. Furthermore, Puyraimond et al. [33] have shown that CAV1 interacts and potentiates the activity of metalloproteinases; other studies have shown the same effect on urokinase receptors leading to the conclusion that CAV1 may serve as an important intercellular signaling molecule that is capable of inducing progression, invasiveness and vascularisation of renal tumors.

A further important fact is that the etiology of most cancers does not reflect alterations in a single gene, but rather the functional loss or induction of a series of key regulatory proteins that, in combination, disrupts the normal regulation of the cell cycle and subsequently leads to uncontrolled cell growth [34]. CAV1 has been recognized to potentiate AKT activity in a variety of model systems. Campbell et al. [35] revealed that when CAV1 is co expressed with pAKT, pmTOR, pS6 or p4E-BP1 within the primary tumor, time to relapse was significantly reduced compared with when either of the individual variables were expressed alone. They have suggested that the co expression of CAV1 and activated components of the AKT/mTOR pathway represents a 'linked molecular signature' that identifies patients with localized RCC that are at high risk of developing metastatic disease that warrants greater postoperative surveillance. Evaluation of the expression status of both CAV1 and mTOR pathway components in these tumors may help to predict tumor response to novel pathway specific therapies, hence allowing appropriate selection of treatment for individual patients. Interestingly, CAV1 has been identified as a molecular target of bortezomib, which has many molecular targets including proteins related to apoptosis, growth signaling/cell cycle heat-shock proteins, and the proteasome pathway. A Phase II trial in patients with advanced RCC showed moderate clinical efficacy for bortezomib [36].

## Conclusion

We were able to show that caveolin-1 protein expression is a predictor of poor disease-free survival in clear cell RCC, suggesting that cell signaling pathways involving caveolin-1 may be of importance in tumor progression. Furthermore, the strength of the association with poor prognosis suggests that CAV1 is likely to be a useful prognostic marker. Therefore, our data encourage further investigations to enlighten the role of CAV1 in tumour progression and to assess its function as prognostic marker for clinical use in serum and/or urine.

## Competing interests

The authors declare that they have no competing interests.

## Authors' contributions

SW designed the study, was part of the acquisition, analysis and interpretation of data and drafted the manuscript. AJS performed the statistical analysis and drafted the manuscript. HB and GV carried out the immunoassays and evaluation. HE was part of the data acquisition. WT evaluated the pathological stages. MAK and JS were responsible for the supervision. All authors read and approved the final manuscript.

## Pre-publication history

The pre-publication history for this paper can be accessed here:

http://www.biomedcentral.com/1471-2490/11/25/prepub

## Supplementary Material

Additional file 1 **Figure S1**. Caveolin-1 staining of peripheral endothelial cells was used as the positive controls. With no staining reaction of the membrane, cytoplasm and nucleus of the tumor cells. Click here for file

Additional file 2 **Figure S2**. Strong membranous Caveolin-1 expression. Click here for file

Additional file 3 **Figure S3**. Association between cytoplasmic CAV1 expression and clinical outcome in all patients (Kaplan-Meier; n = 169) focusing on the individual cellular maximum staining score: The tumor-specific survival of patients with a CAV1 staining score of 4 or 5 (n = 19) in the tumor cell cytoplasm was significantly shorter compared with that of patients with a score of 2-3 (n = 38) and even more 0-1 (n = 112). 5-year tumor specific survival rate were calculated at 26.3%, 60.0%, and 80.1% (p < 0.001, Mantel-Cox). Click here for file

Additional file 4 **Figure S4**. Association between cell membrane CAV1 expression and clinical outcome in all patients (Kaplan-Meier; n = 169) focusing on the individual cellular maximum staining score: The tumor-specific survival of patients with a CAV1 staining score of 2 or 3 (n = 50) in the tumor cell membrane was slightly but insignificantly shorter compared with that of patients with a score of 1 (n = 85) or 0 (n = 34). 5-year tumor specific survival rate were calculated at 61.9%, 72.8%, and 72.7% (p = 0.56, Mantel-Cox). Click here for file
