# Supplementary figures and images for: Caveolin 1 protein expression in renal cell carcinoma predicts survival
Source: BMC Urol. 2011 Dec 7;11:25. doi: 10.1186/1471-2490-11-25 (PMC3266190; doi:10.1186/1471-2490-11-25)

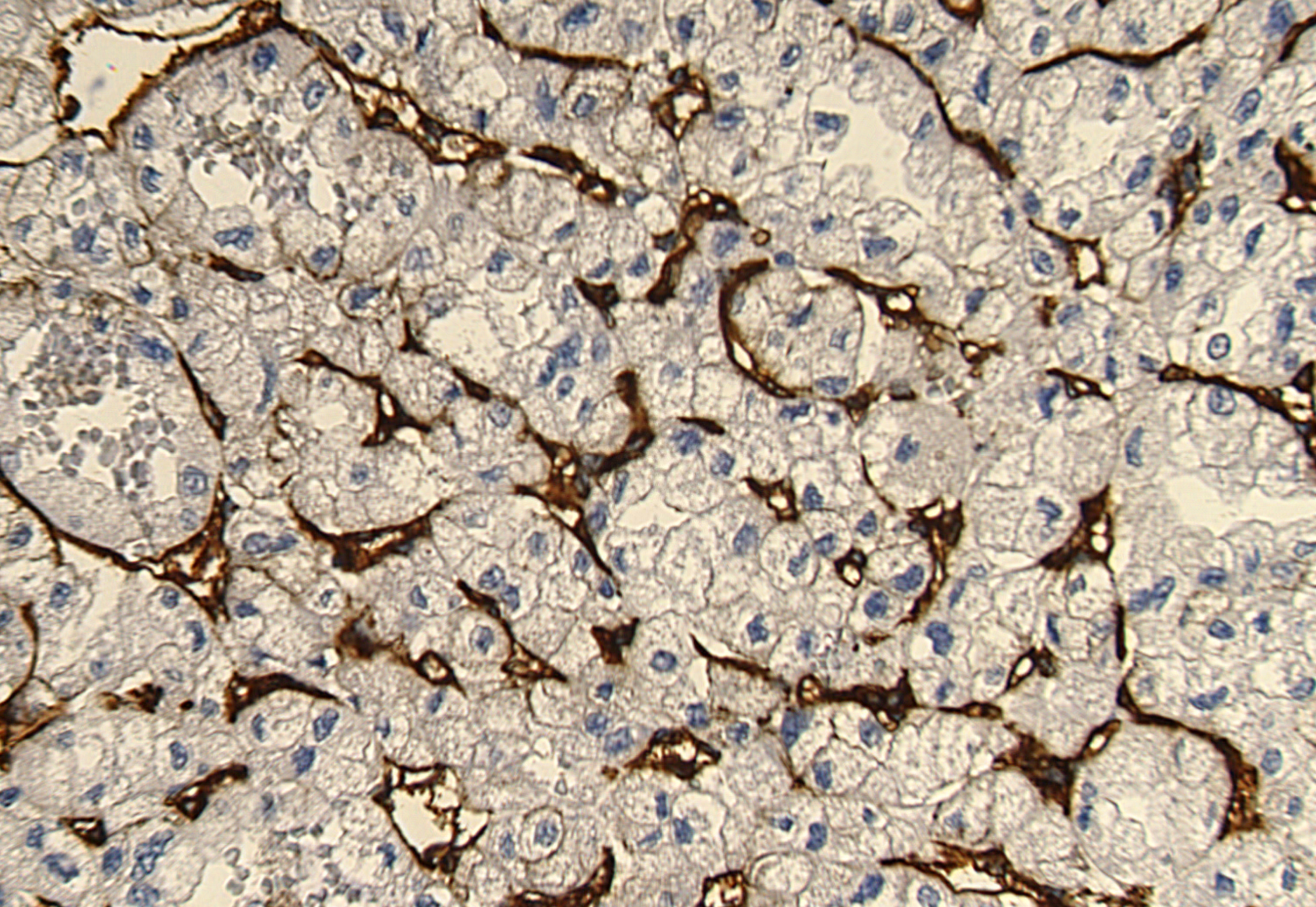

Supplement: Additional file 1 — Figure S1. Caveolin-1 staining of peripheral endothelial cells was used as the positive controls. With no staining reaction of the membrane, cytoplasm and nucleus of the tumor cells. [file 1471-2490-11-25-S1.JPEG]

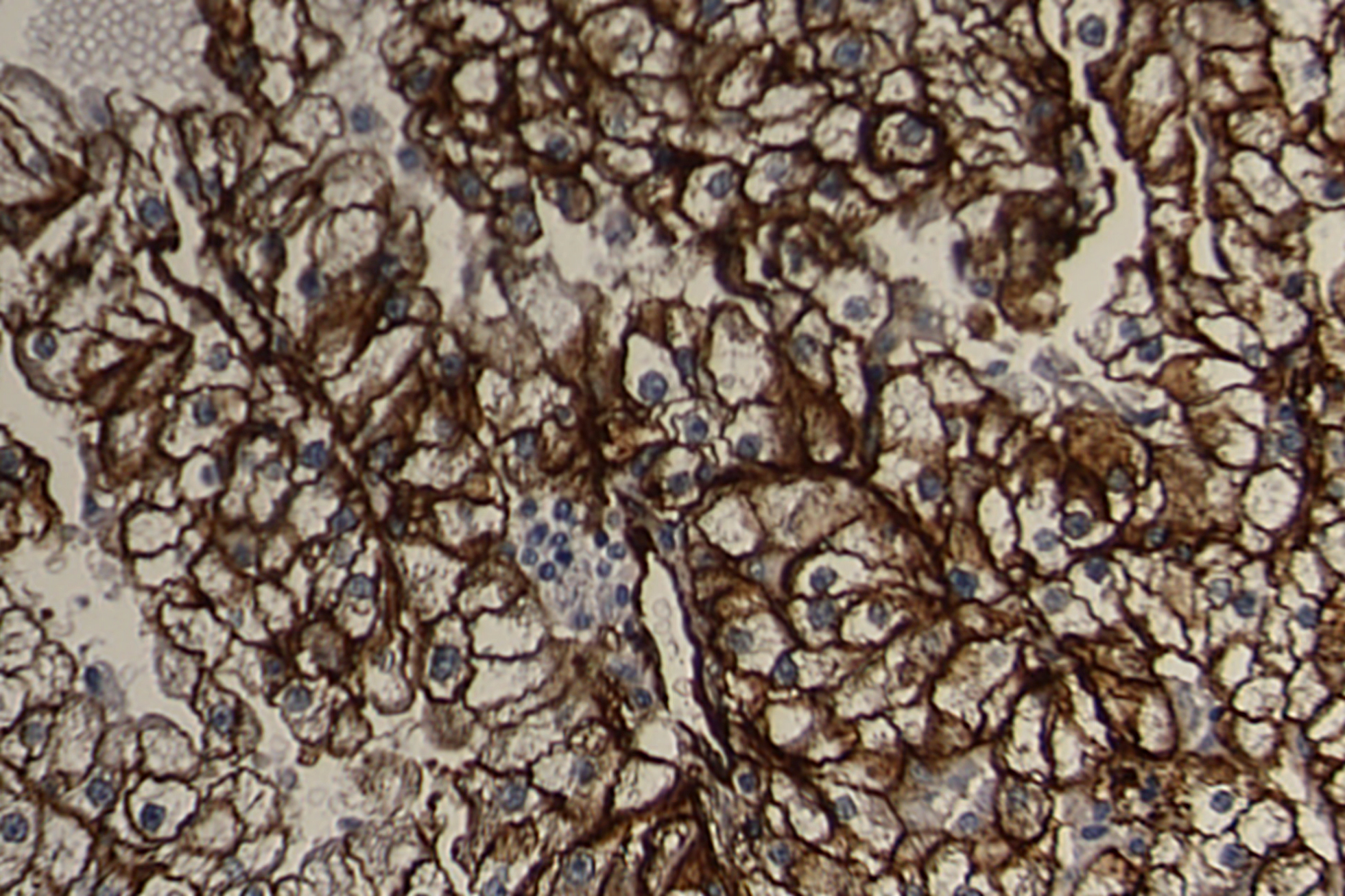

Supplement: Additional file 2 — Figure S2. Strong membranous Caveolin-1 expression. [file 1471-2490-11-25-S2.JPEG]

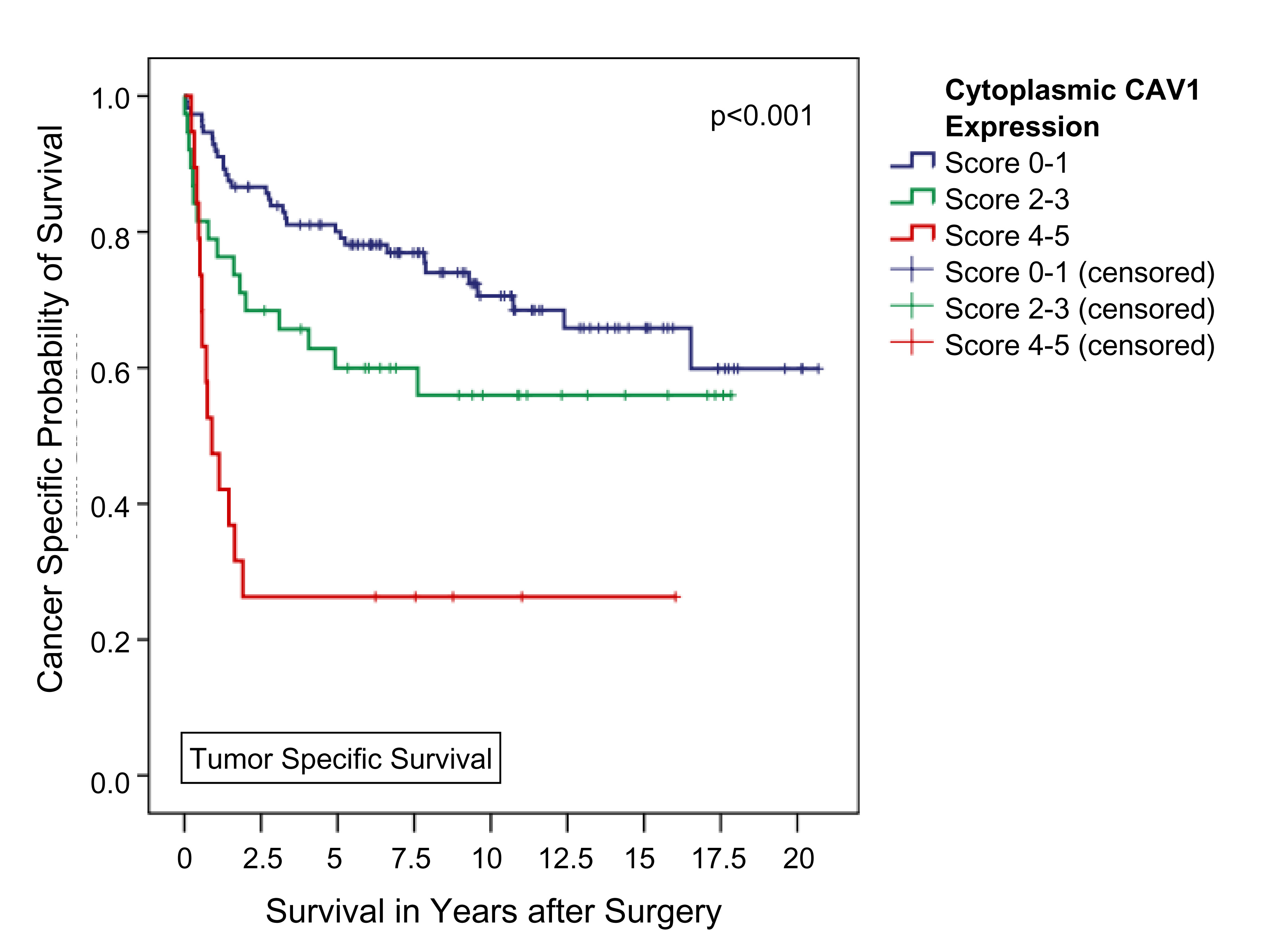

Supplement: Additional file 3 — Figure S3. Association between cytoplasmic CAV1 expression and clinical outcome in all patients (Kaplan-Meier; n = 169) focusing on the individual cellular maximum staining score: The tumor-specific survival of patients with a CAV1 staining score of 4 or 5 (n = 19) in the tumor cell cytoplasm was significantly shorter compared with that of patients with a score of 2-3 (n = 38) and even more 0-1 (n = 112). 5-year tumor specific survival rate were calculated at 26.3%, 60.0%, and 80.1% (p < 0.001, Mantel-Cox). [file 1471-2490-11-25-S3.JPEG]

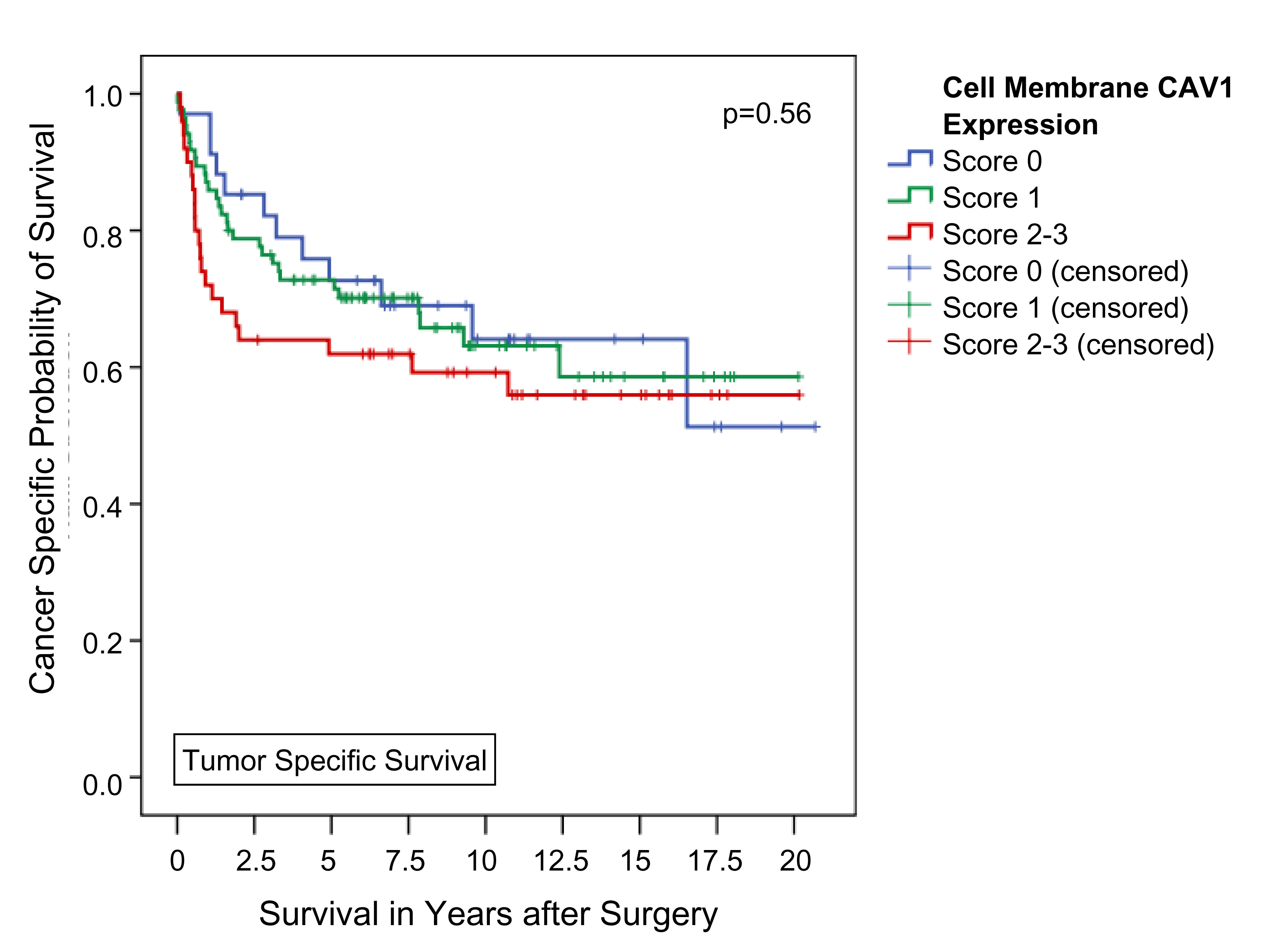

Supplement: Additional file 4 — Figure S4. Association between cell membrane CAV1 expression and clinical outcome in all patients (Kaplan-Meier; n = 169) focusing on the individual cellular maximum staining score: The tumor-specific survival of patients with a CAV1 staining score of 2 or 3 (n = 50) in the tumor cell membrane was slightly but insignificantly shorter compared with that of patients with a score of 1 (n = 85) or 0 (n = 34). 5-year tumor specific survival rate were calculated at 61.9%, 72.8%, and 72.7% (p = 0.56, Mantel-Cox). [file 1471-2490-11-25-S4.JPEG]
